# Dental status and its correlation with polypharmacy and multimorbidity in a Swiss nursing home population: a cross-sectional study

**DOI:** 10.1007/s00784-023-04906-6

**Published:** 2023-03-07

**Authors:** Noemi Anliker, Pedro Molinero-Mourelle, Mariëtte Weijers, Hristina Bukvic, Michael M. Bornstein, Martin Schimmel

**Affiliations:** 1 Department of Reconstructive Dentistry and Gerodontology, School of Dental Medicine, University of Bern, Freiburgstrasse 7, 3010 Bern, Switzerland; 2 Department of Oral Health & Medicine, University Center for Dental Medicine Basel UZB, University of Basel, Basel, Switzerland; 3 Division of Gerodontology and Removable Prosthodontics, University Clinics of Dental Medicine, University of Geneva, Geneva, Switzerland

**Keywords:** Polypharmacy, Multimorbidity, Geriatrics, Oral health, Tooth loss, Dental care for age

## Abstract

**Objective:**

To assess the correlation between oral health status in terms of present teeth, implants, removable prostheses, and polypharmacy and/or multimorbidity in three Swiss nursing homes with affiliated or integrated dental care.

**Methods:**

A cross-sectional study was conducted in three Swiss geriatric nursing homes with integrated dental care. Dental information consisted of the number of teeth, root remnants, implants, and presence of removable dental prostheses. Furthermore, the medical history was assessed in terms of diagnosed medical conditions and prescribed medication. Age, dental status, polypharmacy, and multimorbidity were compared and correlated using *t*-tests and Pearson correlation coefficients.

**Results:**

One hundred eighty patients with a mean age of 85.5 ± 7.4 years were included of which a portion of 62% presented with multimorbidity and 92% with polypharmacy. The mean number of remaining teeth and remnant roots were 14.1 ± 9.9 and 1.0 ± 3.1, respectively. Edentulous individuals comprised 14%, and over 75% of the population did not have implants. Over 50% of the included patients wore removable dental prostheses. A negative correlation with statistical significance (*p* = 0.001) between age and tooth loss (*r* = − 0.27) was observed. Finally, there was a non-statistically correlation between a higher number of remnant roots and specific medications linked to salivary dysfunction; specifically antihypertensive medication and central nervous system stimulants.

**Conclusion:**

The presence of a poor oral health status was associated with polypharmacy and multimorbidity among the study population.

**Clinical relevance:**

Identifying elderly patients in need of oral healthcare in nursing homes is a challenge. In Switzerland, the collaboration of dentists and nursing staff is still improvable, but is urgently needed due to the demographic changes and raising treatment demand of the oldest portion of the population.

## Introduction

Average life expectancy steadily increased worldwide over the last decades and currently reaches almost 85 years in several industrialized countries [1, 2]. Among others, this finding is mostly related to significant improvements in the standard of living and improvement in healthcare. Concomitantly, the drop in fertility rates seen after the “baby boom” generation has led to a drastic demographic change with a steadily growing population of old and very old individuals leading to an inversion of the classic age pyramid [2].

Currently, in Switzerland, 60% of the population are aged between 40 and 64 years old, and a further 1.6 million (19%) are 65 + years. This shift is expected to increase further [3]. The Swiss Federal Statistical Office (SFSO) estimates that by the year 2050, 2.7 million Swiss residents, a fourth (26%) of the entire current Swiss population, will be 65 years or older [3]. With increasing age, also the percentage people living in a long-term care homes increases [1]. In 2010, 125,000 aged Swiss residents were dependent on some sort of care (nursing home or care at home) [4]. By 2030, this number could increase to 182,000 people (a plus of 46%) [4].

One of the consequences of an aging population is the prevalence of multimorbidity, defined as the presence of at least two chronic medical conditions in an individual [5, 6]. This multimorbidity is often accompanied by polypharmacy, defined as the concomitant daily use of five or more drugs by the same individual [7]. In Switzerland, the prevalence of polypharmacy is 61% of the population between 65 and 81 years of age; taking 5 or more drugs [8].

Notably, in 2017, over 122,000 elderly people lived permanently in nursing homes in Switzerland [9, 10], and these people often suffer from multimorbidity and polypharmacy [6]. Besides age, other factors contributing to multimorbidity have also been discussed, such as socioeconomic status or nutritional status [11]. Although oral health may not be a priority in this population group, it should be taken into consideration, since many medical conditions and medications are closely related to oral health. Drugs have been directly or indirectly associated with the development of caries, periodontal diseases, oral mucosal pathologies, difficulties in mastication, xerostomia, aspiration pneumonia, or complications related to dentures [8, 12–16]. A potential risk for oral health of nursing home residents is the dependency on help for various activities of daily living (ADL) ([17]), and therefore, seniors are often dependent on help to maintain their oral health [11, 18]. The oral health of elderly individuals living in nursing homes remains poor compared to aged people living independently [11, 17, 19], since many dependent elders need longer treatment time due to the age health-related problems and their reduced mobility.

Although the relationship between oral health and general health has been widely investigated for the general population, there is a lack of evidence evaluating the relationship between oral health status, polypharmacy, and multimorbidity in elderly nursing homes with affiliated dental care [20]. Therefore, the aim of the present study was to assess the correlation between oral health status in terms of present teeth, implants, and removable prostheses and (a) polypharmacy and/or (b) multimorbidity in a representative geriatric Swiss population comprising of three nursing homes with affiliated or integrated dental care. The null hypothesis for this study was that there is no correlation between oral health and polypharmacy and/or multimorbidity.

## Material and methods

### Study design

The present study was designed as a cross-sectional study adhering to STROBE guidelines [21] and in compliance with the Declaration of Helsinki of 2013 [22]. Formal approval of the study protocol was granted by the local ethical committee of the Canton of Bern (KEK, Nr.:reg2016-00,244). All patients or their legal counsel signed an informed consent that their dental and medical history be used anonymously for research.

### Study population

The study population consisted of a geriatric Swiss cohort. Information was gathered based on clinical records of medical and dental history files of three affiliate nursing homes with the Department of Reconstructive Dentistry and Gerodontology, School of Dental Medicine, University of Bern. Affiliate clinics had integrated dental care units as a part of the health care service. Between 2016 and 2020, nine specialized and calibrated prosthodontists from the same department visited the centers once per week or per 2 weeks and those individuals with dental care needs were treated. In one center, a permanent dental unit including a full-equipped dental unit equipped with a chairside X-ray machine and panoramic X-ray device was available. For the other two clinics, portable dental units with a limitation to clinical examination and basic dental treatment were present.

Inclusion criteria were individuals with dental and medical records over 65 years old with diagnosed polypharmacy and/or multimorbidity as defined above and with enough clinical information about their dental state within the records. The exclusion criteria were lack of related records in the medical and dental history and states.

### Assessed parameters

Information was retrospectively collected by a single calibrated and independent investigator from the same department, who did not contribute to the clinical examinations. Based on the general anamnesis, medical files, and dental clinical history, the assessed information was compiled as follows:

- General health information: Names of the medical conditions diagnosed by physicians were assessed. The co-occurrence of two or more chronic medical conditions in one person was considered as multimorbidity [5]. Prescribed drug names were registered except posology. Simultaneous administration of five or more multiple medications to the same patient was qualified as polypharmacy [7]. Medication was assorted into groups according to Fitzgerald et al. and those who are known to be linked to salivary dysfunction were further investigated [23–25]. Namely the following drug groups: antihypertensive medication (AHM), psychiatric medication (PM), opioids (O), antihistamine medication (AHiM), sedative medication (SM), benign prostatic hyperplasia medication (PHM), central nervous system stimulants (CNS), anticonvulsive medication (ACM) [23–25].
- Dental state: Number of present teeth, root remnants, implants, and presence of removable dental prostheses were recorded. Removable dental prostheses were considered as removable complete dentures (CD) and removable partial dentures (RPD), including tooth and implant-assisted RPDs or tooth or implant-supported overdentures. The definition of dental status was based on the number of teeth present [14]. Heavily decayed teeth and/or non-restored root remnants (i.e., no filling or root canal treatment) were considered as an indicator for impaired dental state/oral health.

### Statistical analysis

For this study, the sample was divided into age groups (5-year groups) and continuous variables were expressed as means and standard deviations (SD) and categorical variables are described as numbers and percentages (%). The Kolmogorov–Smirnov test was used to evaluate the distribution of the obtained results. Due to the non-normal distribution and to compare means of continuous variables in 2 different groups, the independent samples *t*-test was used. In conditions of heterogeneous variances, the Welch correction was used. Mann–Whitney test was used to compare the variable distributions in two independent groups if one of them had a limited sample size (*n* < 30). Finally, the Pearson correlation coefficient was estimated to evaluate the linear association between continuous variables (i.e., number of teeth and age). The partial correlation was estimated when the influence of third variables is suspected (i.e., number of teeth and roots, controlling for age).

The statistical analysis was performed by using a statistical software program (SPSS V25.0; IBM Corp, Armonk, NY) and *p* values smaller than 0.05 were considered to be statistically significant.

## Results

A total sample of 180 patients was included with a proportion of 75% (135) for females and a total mean age of 85.5 ± 7.4 years ranging from 67 to 100 years (Table 1). Considering the medical conditions, 62% of the individuals presented with multimorbidity, and 92% were diagnosed as polymedicated. The mean number of medications per patient was 12.1 ± 5.6. The most frequent prescribed drugs associated with hyposalivation were antihypertensives (62%) followed by psychiatric drugs (54%), sedatives (51%), and opioids (26%). The rate of any other type of medication did not exceed 10%.

**Table 1:** Age groups according to sex

	Age group															
	Total		65–70		70–75		75–80		80–85		85–90		90–95		95–100	
	*N*	%	*N*	%	*N*	%	*N*	%	*N*	%	*N*	%	*N*	%	*N*	%
Total	180	100	5	100	17	100	21	100	30	100	48	100	42	100	17	100
Male	45	25.0	2	40.0	4	23.5	7	33.3	9	30.0	9	18.8	6	14.3	8	47.1
Female	135	75.0	3	60.0	13	76.5	14	66.7	21	70.0	39	81.3	36	85.7	9	52.9

Regarding the dental status, the mean number of teeth present was 14.1 ± 9.9, and the mean number of root remnants was 1.0 ± 3.1. A total of 14% of the patients were edentulous, and 30% had at least 20 teeth. For the restorative evaluation, more than 75% of the included patients did not present with implants, and 52% of the observed sample had removable dental prostheses (Tables 2 and 3).

**Table 2:** Number of teeth, roots, and implants by age group

		Age group							
		Total	65–70	70–75	75–80	80–85	85–90	90–95	95–100
Teeth present	Patients	146	4	15	17	27	37	32	14
	Mean	14.1	17.3	19.4	16.3	17.1	11.2	13.3	8.4
	SD	9.9	11.7	10.8	11.1	7.9	10.2	8.7	8.4
	Minimum	0.0	0.0	0.0	0.0	0.0	0.0	0.0	0.0
	Maximum	32.0	25.0	32.0	30.0	28.0	29.0	26.0	25.0
	Percentile 25	5.0	10.0	12.0	4.0	11.0	0.0	6.5	2.0
	Median	15.0	22.0	21.0	20.0	18.0	9.0	13.5	5.0
	Percentile 75	23.0	24.5	29.0	24.0	24.0	21.0	20.0	15.0
Root remnants present	Patients	146	4	15	17	27	37	32	14
	Mean	1.02	0.50	0.07	1.00	0.15	1.65	1.75	0.57
	SD	3.08	1.00	0.26	1.97	0.46	4.95	3.31	1.09
	Minimum	0.00	0.00	0.00	0.00	0.00	0.00	0.00	0.00
	Maximum	21.00	2.00	1.00	6.00	2.00	21.00	16.00	3.00
	Percentile 25	0.00	0.00	0.00	0.00	0.00	0.00	0.00	0.00
	Median	0.00	0.00	0.00	0.00	0.00	0.00	0.00	0.00
	Percentile 75	0.00	1.00	0.00	1.00	0.00	0.00	2.00	1.00
Implants present	Patients	149	4	15	18	27	39	32	14
	Mean	0.40	0.25	0.27	0.56	0.48	0.46	0.31	0.29
	SD	1.08	0.50	0.80	1.46	1.05	1.12	1.15	0.73
	Minimum	0.00	0.00	0.00	0.00	0.00	0.00	0.00	0.00
	Maximum	6.00	1.00	3.00	6.00	3.00	4.00	6.00	2.00
	Percentile 25	0.00	0.00	0.00	0.00	0.00	0.00	0.00	0.00
	Median	0.00	0.00	0.00	0.00	0.00	0.00	0.00	0.00
	Percentile 75	0.00	0.50	0.00	0.00	0.00	0.00	0.00	0.00

**Table 3:** Presence of removable prostheses by age group (*n* = patients)

	Age group															
	Total		65–70		70–75		75–80		80–85		85–90		90–95		95–100	
	*N*	%	*N*	%	*N*	%	*N*	%	*N*	%	*N*	%	*N*	%	*N*	%
Total	172	100.0	5	100.0	16	100.0	19	100.0	30	100.0	47	100.0	39	100.0	16	100.0
No	82	47.7	4	80.0	10	62.5	11	57.9	12	40.0	21	44.7	20	51.3	4	25.0
Yes	90	52.3	1	20.0	6	37.5	8	42.1	18	60.0	26	55.3	19	48.7	12	75.0

**Fig. 1:**
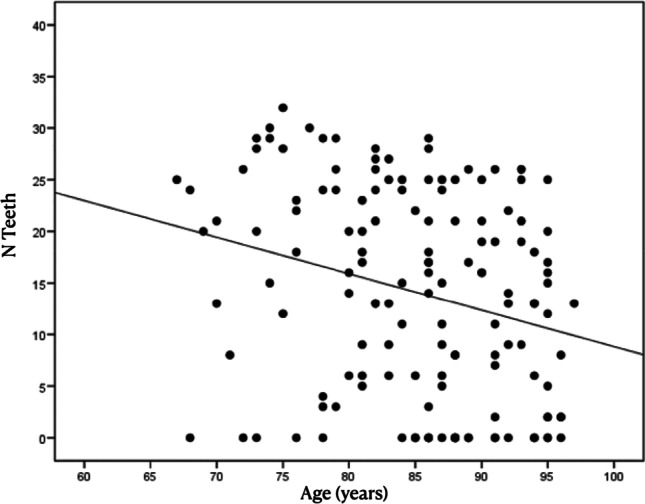
Number of teeth present decreasing with age of included patients. r=-0.27

**Fig. 2:**
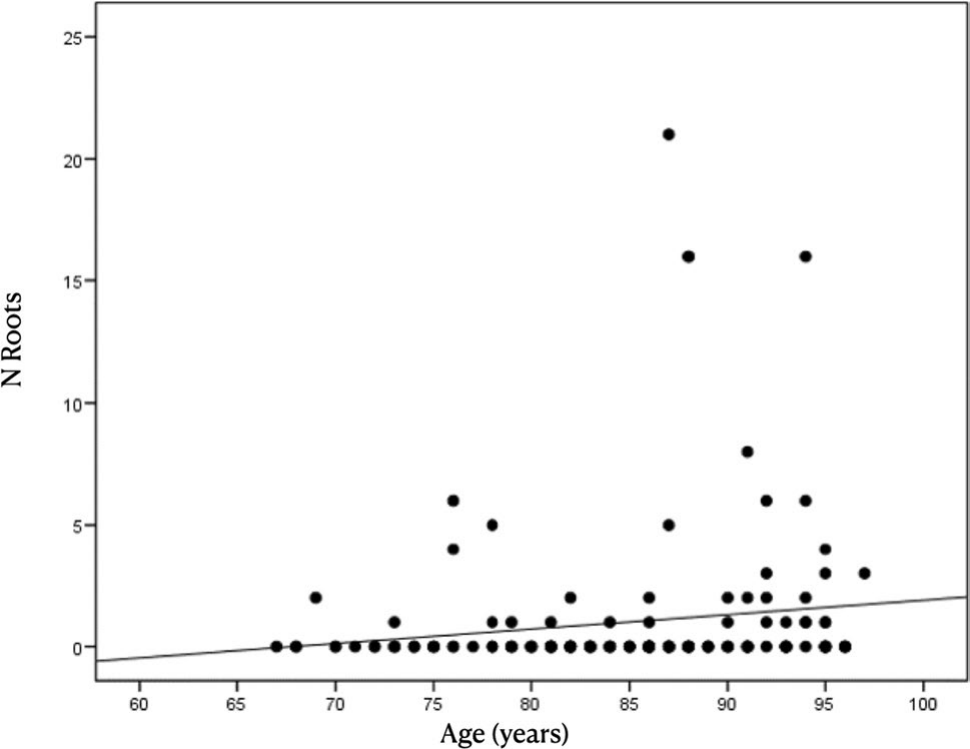
Increase in number of root remnants with age of included patients. r=0.14

**Fig. 3:**
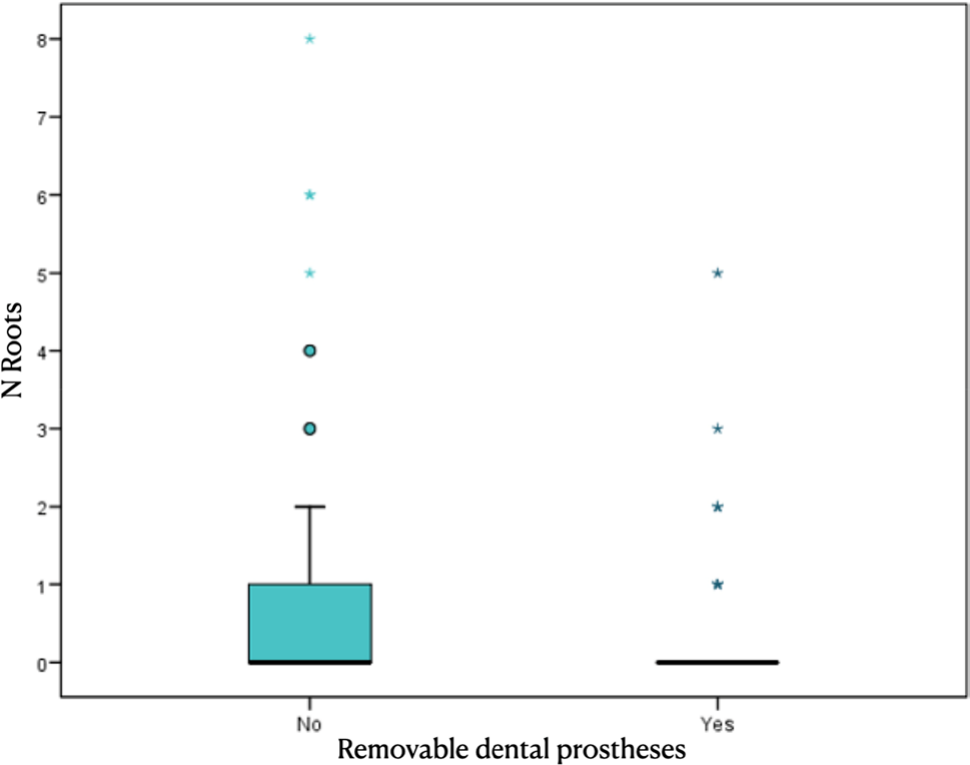
Lower prevalence of root remnants in removable dental prostheses wearers. Removable dental prostheses; p=0.035

Concerning the number of teeth in regard to multimorbidity and polypharmacy, no differences in the means (or distributions) (*p* = 0.253) were found. Concurrently, the number of root remnants did not show a significant correlation compared with multimorbidity (*p* = 0.547). For the correlation with age, the results obtained showed a negative correlation (*r* = − 0.27) with respect to tooth loss, which means a progression with the age of the patient (*p* = 0.001) (Fig. 1). In addition, the number of root remnants present (*r* = 0.14) increased with age (*p* = 0.087) (Fig. 2). Considering the number of teeth, a relation between the number of teeth and remnant roots (*r* = − 0.15; *p* = 0.069) was found. The RDP wearer evaluation showed an association between an RDP and a lower number of remnant roots. The mean number of root remnants in patients with RDP was 0.5 ± 2.0 versus 1.6 ± 3.9 in subjects without RDP, which was found to be statistically significant in the *t*-test (*p* = 0.035) as well in the Mann–Whitney test (*p* = 0.026) (Table 4 and Fig. 3).

**Table 4:** Presence of removable prostheses (Yes/No) by number of root remnants (*n* = patients)

	Removable dental prostheses		
	Total	No	Yes
Patients (*N*)	145	70	75
Mean (roots)	1.03	1.60	0.49
Standard deviation (roots)	3.09	3.89	1.98
Minimum (roots)	0.00	0.00	0.00
Maximum (roots)	21.00	21.00	16.00
Percentile 25 (roots)	0.00	0.00	0.00
Median (roots)	0.00	0.00	0.00
Percentile 75 (roots)	0.00	1.00	0.00

Finally, a correlation between the dental status and medication taken was observed, although not reaching statistical significance, specific medication groups are known to be linked to salivary dysfunction [24]. The number of root distribution was higher in patients with AHM (*p* = 0.087) and CNS (*p* = 0.063) type of medication (Table 5).

**Table 5:** Number of root remnants by type of medication (*n* = patients)

	Root remnants	*N*	Mean	SD	Minimum	Maximum
Antihypertensive	Total	146	1.02	3.08	0.00	21.00
	No	54	0.56	1.3	0.00	6.00
	Yes	92	1.29	3.74	0.00	21.00
Psychiatric medication	Total	146	1.02	3.08	0.00	21.00
	No	68	0.87	2.43	0.00	16.00
	Yes	78	1.15	3.57	0.00	21.00
Opioids	Total	146	1.02	3.08	0.00	21.00
	No	110	0.8	2.13	0.00	16.00
	Yes	36	1.69	4.96	0.00	21.00
Antihistamine	Total	146	1.02	3.08	0.00	21.00
	No	132	1.12	3.23	0.00	21.00
	Yes	14	0.07	0.27	0.00	1.00
Sedative	Total	146	1.02	3.08	0.00	21.00
	No	74	0.96	2.87	0.00	16.00
	Yes	72	1.08	3.31	0.00	21.00
Prostatic hyperplastic mediation	Total	146	1.02	3.08	0.00	21.00
	No	140	0.95	2.87	0.00	21.00
	Yes	6	2.67	6.53	0.00	16.00
Nervous system stimulants	Total	146	1.02	3.08	0.00	21.00
	No	136	1.01	3.17	0.00	21.00
	Yes	10	1.2	1.69	0.00	5.00
Anticonvulsive medication	Total	146	1.02	3.08	0.00	21.00
	No	133	1.05	3.2	0.00	21.00
	Yes	13	0.69	1.55	0.00	5.00

## Discussion

The current study investigated a potential correlation between oral health in terms of teeth, root remnants, implants, and removable dental prostheses with polypharmacy and multimorbidity in a geriatric Swiss population consisting of three nursing homes with affiliated dental care. Considering the obtained results, the null hypothesis was accepted since no statistically significant correlation between dental state and multimorbidity or medication was found.

It is known that tooth loss progresses with age [17, 20, 26] and in this sense, the obtained results corroborate the literature since the present sample included elderly 146 patients having still teeth present in a sample of 180. It could be observed that advanced age patients showed a greater presence of root remnants (*p* = 0.087) and with certain drug groups, specifically AHM (*p* = 0.087) and CNS (*p* = 0.063). In contrast, RDP wearers had significantly fewer root remnants (*p* = 0.026). As the extraction of root remnants should usually be performed before manufacturing a removable dental prosthesis, this fact could contribute to the lower number of remaining root remnants. In the present study, the mean age of included patients was 85.5 years representing a sample of the oldest generation of the reported population which is scarce in epidemiological studies based—especially also in Switzerland [19]. Considering the dental status, the proportion of edentulous patients was by approximately 5% lower as reported in Brändli-Holzer et al., but higher when compared with Schneider et al., which have also investigated a Swiss population [17, 19].

Relations and effects between oral health and general health status are generally accepted [13, 27, 28]. The 2016 World Dental Federation (FDI) oral health definition states “the fundamental component of health and physical and mental wellbeing” as part of the state of health, which implies that oral health is integrated in general health [14]. On the same lines, the World Health Organization (WHO) frail older individual’s statement includes that frail elderly more often suffer from oral conditions which can be related with the number of teeth present, and therefore, these patients exhibit nutritional deficiencies and prosthetic rehabilitations with higher prevalence in comparison to their non-infirm contemporaries [14, 29].

Considering the high percentage of multimorbidity (62%) and polypharmacy (92%) reported in the present study, no differences in the mean number of teeth or remnant roots were found (*p* = 0.253). Comparing our results with a similar study with a younger population sample by Bopp and Holzer [65 to 81 years old (61%)], our study found a higher prevalence of patients exhibiting polypharmacy. However, when our results were assessed, the lack of a control group should be considered as a study limitation.

In this investigation, untreated root remnants were distinguished from teeth that still maintain a crown and they were considered as indicators of poor oral health. A non-significant higher number of root remnants with AHM and CNS intake could be observed. It is important to mention that age alone is no cause of promoting hyposalivation [30]. A recent study in a population of Japanese elderly found a correlation between a higher number of decayed teeth and dementia [20]. Certain forms of dementia are treated with CNS. Nevertheless, exact dementia mechanisms remain unclear, and considering that related CNS medications are involved in hyposalivation, this might lead to an increased presence of tooth decay [24, 25, 31].

Considering the overall obtained results, clinicians should keep in mind that elderly individuals, especially those living in long-term care homes, often present with a multitude of diseases and concomitant medication, leading to a rise in treatment complexity. Although the present sample did not exhibit a high number of dental implants, this treatment option should be carefully considered since implants may become a health hazard in elders dependent on care due to progressive general and oral aging-related problems and/or insufficient dental implant care. Therefore, less invasive prosthodontic options that can be easily managed should be recommended taking into account the systemic conditions and estimated life expectancies of the patients treated [32].

Some medications can support or even accelerate tooth decay [12]. This may lead to a worse oral health status in elderly in terms of more retained and/or decayed root remnants. Furthermore, the effect of impaired general health on oral health must be differentiated into direct negative effects of the disease or indirect sequelae via the medication taken for a specific disease (i.e., hyposalivation, syrups with sugar) [24, 30, 31, 33, 34]. When the study limitations are considered, the cross-sectional design and a lack of control group should be mentioned. Furthermore, the patient’s sample is relatively small compared to other epidemiological studies, and examiner’s blinding was not possible during the dental evaluation. A detailed overview of the dental and prosthetic status was not obtained, and therefore, a functional evaluation could not be performed. Different study design, i.e., with follow-up and larger patient samples, could result in more meaningful outcomes.

## Conclusions

There was an association between the presence of poor oral health status and polypharmacy and multimorbidity status among the study population evaluated. Further preventive and cooperative programs between nursing homes and dental practitioners in Switzerland are needed especially with regard to the ongoing demographic changes and respective dental treatment demands.

## Data Availability

The data that support the findings of this study are partially available on request from the corresponding author.
